# Multi-omics analysis reveals the effects of prenatal nutrition on carcass-related tissues in beef cattle

**DOI:** 10.1038/s41598-025-31856-8

**Published:** 2025-12-13

**Authors:** Guilherme Henrique Gebim Polizel, Ángela Cánovas, Wellison J. S. Diniz, German D. Ramírez-Zamudio, Saulo da Luz e Silva, Carl R. Dahlen, Arícia Christofaro Fernandes, Barbara Carolina Teixeira Prati, Édison Furlan, Gabriela do Vale Pombo, Miguel Henrique de Almeida Santana

**Affiliations:** 1 Department of Animal Science, GOPec, Faculty of Animal Science and Food Engineering, University of São Paulo, Av. Duque de Caxias Norte, 225, Pirassununga, 13635-900 SP Brazil; 2 Department of Animal Biosciences, University of Guelph, 50 Stone Road East, Guelph, ON Canada; 3 Department of Animal Sciences, College of Agriculture, Auburn University, Auburn, AL USA; 4 Department of Animal Sciences, Center for Nutrition and Pregnancy, North Dakota State University, Fargo, ND USA

**Keywords:** Fat, Lipid metabolism, Maternal nutrition, Meat, Muscle, Systems biology, Biochemistry, Physiology, Zoology

## Abstract

This study evaluated the long-term metabolic effects of prenatal nutrition in Nellore bulls. Pregnant cows (*n* = 126) received mineral supplementation only (NP), protein–energy supplementation during the last trimester (PP), or supplementation throughout pregnancy (FP). At slaughter, longissimus (muscle and meat) and subcutaneous fat samples from the offspring were collected for transcriptomics and metabolomics analyses. Data were reduced using Weighted Gene Co-expression Network Analysis, followed by functional enrichment, and then integrated via Spearman’s correlations and holistic pathway analysis. Distinct molecular patterns emerged across prenatal nutrition treatments, although all groups influenced energy metabolism and cellular processes. The NP group was strongly associated with protein and lipid metabolism, highlighted by PPAR and sphingolipid signaling pathways, and key hub components including *CNOT4* and tryptophan. In contrast, PP and FP groups were more closely linked to immune function, stress resilience, with enrichment of NF-kB signaling, cortisol synthesis, and hub components including *TIE1*, *YWHAZ*, carnitine, and glutaconylcarnitine. Shared transcriptome–metabolome modules between groups displayed inverse correlations, suggesting potential antagonistic effects driven by maternal diet. Overall, these results indicate that prenatal nutrition shapes key metabolic processes in muscle, meat, and fat, offering insights to enhance meat quality and production through maternal feeding strategies.

**Supplementary Information:**

The online version contains supplementary material available at 10.1038/s41598-025-31856-8.

## Introduction

The Americas account for 39.8% of the global cattle population, encompassing both dairy and beef cattle, making the region a significant hub for cattle farming. Notably, Brazil contributes 14.7% of the world’s total cattle population, highlighting its central role in global cattle production^1^. In tropical and subtropical regions, cow-calf systems are predominantly managed under extensive or semi-extensive grazing conditions, characterized by significant variability in forage resources^2^. Regardless of the management strategy, adequate prenatal nutrition for the cow is essential for optimal fetal development, and positively influences the offspring’s productive and reproductive performance throughout life^3–6^.

In beef cattle production, where meat quality and quantity are priorities, one of the main objectives of the system is to promote the proper development of skeletal muscle and adipose tissues. Since hyperplasia of skeletal muscle fibers occurs exclusively during fetal life^7^, maternal nutrition and the intrauterine environment strongly influence the animal’s potential for meat yield. The second trimester of gestation is a critical period for muscle formation^8^. The final trimester of gestation is marked by rapid fetal growth^9^, emphasizing muscle hypertrophy and adipogenesis, which directly affect the composition of final retail products^10,11^. As a result, most studies investigating the effects of maternal nutrition on offspring development focus on the second and third trimesters of gestation^8,12^. However, the first trimester is also a critical period, characterized by placental establishment and vascularization, as well as fetal organogenesis^13,14^, with potential repercussions for the offspring’s metabolic outcomes in postnatal life^15^. Despite its importance, this early window remains underexplored in beef cattle, and it is unclear whether nutritional interventions restricted to later stages are sufficient to offset potential programming effects that occur during early gestation.

Understanding and optimizing the complex processes of development requires advanced tools capable of deciphering the underlying molecular and cellular mechanisms that are responsible for observed effects. A multi-omics integration approach, in particular, has emerged as a powerful approach for comprehensively studying multiple layers of molecular data simultaneously in important tissues of beef cattle production^16^. Integrating transcriptomic data with metabolomic data and functional genomics information, for example, enables the identification of gene-metabolite interplay and the metabolic pathways involved with the both omics concurrently^17^. In our recent study^18^, through transcriptomics-metabolomics integration, we observed that prenatal nutrition impacted hepatic pathways related to energy and protein metabolism, immune system regulation, and other regulatory factors. Building on this, the present study extends the focus beyond the liver to muscle, meat, and fat, which are directly linked to beef quality traits. Moreover, by comparing supplementation restricted to the last trimester with supplementation maintained throughout pregnancy, we address whether the effects of early gestation nutrition persist into later life or can be compensated by later interventions.

The hypothesis of this study is that different prenatal nutritional strategies have long-term effects impacting the longissimus thoracis (muscle and meat), as well as subcutaneous fat tissue transcriptomic-metabolomic interactions. The objectives of the present study were fourfold: (1) to evaluate whether the protein-energy supplementation throughout gestation or during the third trimester of gestation impacted the muscle transcriptome co-expression networks, as well as meat and subcutaneous fat metabolome co-abundance networks, in bulls during the finishing phase; (2) to evaluate the association between significant co-expressed genes and co-abundant metabolites and their involvement in metabolic pathways; (3) to assess the Spearman’s correlations between the muscle transcriptome and metabolome (meat and subcutaneous fat); (4) to perform an integrated analysis between transcriptome and metabolome to assess the impact of prenatal nutrition on metabolic pathways associated with both omics.

## Methods

### Experimental design

All experimental protocols were approved by the School of Animal Science and Food Engineering (FZEA-USP) committee under the project reference number 1,843,241,117. The experimental protocols were conducted in accordance with the relevant guidelines and regulations. The study design and analysis conform to the ARRIVE recommendations for animal research (https://arriveguidelines.org). All animals used in this experiment were provided by the campus of the FZEA-USP.

A total of 126 Nellore cows (average age 3.52 ± 1.43 years) and their offspring sourced from the FZEA-USP campus. The cows were artificially inseminated with semen from four sires, and pregnancy was confirmed after 30 days. To create balanced groups, the cows were assigned to one of the three nutritional groups based on age, body weight (BW), and body condition (*n* = 42 cows per group). All groups were kept in pastures (*Urochloa brizantha* cv. Marandu) with access to water and feed supplements. The study compared three prenatal feeding strategies: the NP (Not Programmed) group, serving as the control, received only mineral supplements at 0.03% of their BW daily throughout pregnancy; the PP (Partial Programming) group received protein and energy supplementation at 0.3% of their BW daily during the third trimester (from day 190 of pregnancy until calving); and the FP (Full Programming) group received the same protein and energy supplementation from pregnancy confirmation until delivery. All three groups of cows received a base level of mineral supplementation (0.03% of BW daily), which was incorporated into the protein-energy supplement given to the PP and FP groups (as detailed in Table 1).

**Table 1:** Composition and nutritional content of supplements provided to dams during pregnancy.

Ingredients	Mineral supplement	Protein-energy supplement
Corn (%)	35	60
Soybean meal (%)	-	30
Dicalcium phosphate (%)	10	-
Urea 45% (%)	-	2.5
Salt (%)	30	5
Minerthal 160 MD (%)*	25	2.5
Total digestible nutrients (%)	26.76	67.55
Crude protein (%)	2.79	24.78
Non-protein nitrogen (%)	-	7.03
Acid detergent fiber (%)	1.25	4.76
Neutral detergent fiber (%)	4.29	11.24
Fat (%)	1.26	2.61
Calcium (g/kg)	74.11	6.2
Phosphorus (g/kg)	59.38	7.24

*Mineral premix composition (Minerthal company): Calcium = 8.6 g/kg; Cobalt = 6.4 mg/kg; Copper = 108 mg/kg; Sulfur = 2.4 g/kg; Fluorine = 64 mg/kg; Phosphorus = 6.4 g/kg; Iodine = 5.4 mg/kg; Manganese = 108 mg/kg; Selenium = 3.2 mg/kg; Zinc = 324 mg/kg; Sodium monensin = 160 mg/kg^19^.

The nutritional quality of the *Urochloa brizantha* cv. Marandu pastures were similar across all groups throughout the pregnancy period. A detailed examination of pasture conditions, as well as the phenotypic and metabolic effects of the NP, PP, and FP treatments on the cows, is provided in Schalch Junior et al.^20^.

After calving, the protein-energy supplementation given during the prenatal period was stopped. All calves, regardless of their dams’ prenatal treatment, received the same health protocols and nutritional care and were kept together until weaning at approximately 240 days (± 28 days). Throughout this time, the cows continued to receive the same mineral supplementation (0.03% of body weight) as they had during pregnancy and were kept in extensive *Urochloa brizantha* cv. Marandu pastures.

### Post-weaning and finishing managements

After weaning, the calves were separated by sex and managed under the same nutritional program until they reached approximately 570 days of age (± 28 days), regardless of their prenatal treatment. During this period, young bulls received different supplements depending on the season: an energy-rich supplement (67.55% TDN, 24.78% CP, 11.24% NDF, 2.61% Fat, at 0.3% of BW) during the dry season (winter), and a protein-rich supplement (53.15% TDN, 30.03% CP, 9.14% NDF, 1.65% Fat, at 0.1% of BW) during the wet season (summer). From birth until 570 days old (± 28 days), the young bulls had access to *Urochloa brizantha cv. Marandu* pastures and free access to water.

A total of 63 bulls (22 NP, 20 PP, and 21 FP) began the finishing phase at about 570 ± 28 days of age and were slaughtered at approximately 676 ± 28 days. Mean slaughter weights and ages were 591.2 ± 40.05 kg (678 ± 29 days) for NP, 602.6 ± 49.65 kg (676 ± 29 days) for PP, and 597.4 ± 51.06 kg (675 ± 28 days) for FP. During the finishing phase, they received three different diets: an initial 15-day adaptation diet, followed by a 35-day diet; and finally, a 56-day finishing diet. Specific data about the composition of finishing phase diets are detailed in Dias et al.^21^. The bulls were slaughtered at the FZEA/USP school slaughterhouse, located about 500 m from the feedlot. Slaughter and carcass processing followed the Brazilian Ministry of Agriculture, Livestock, and Supply (MAPA) guidelines (Normative Instruction No. 9 of 2004). Meat quality data for these bulls are available in Fernandes et al.^6^.

### Tissue sample collection

After slaughter, samples of the longissimus thoracis muscle (between the 12th and 13th ribs) were collected from all bulls for transcriptomic analysis. These samples were collected within 15 min of slaughter, immediately snap-frozen in liquid nitrogen, and stored at −80 °C. Here, the term muscle refers specifically to tissue collected at the moment of slaughter, before any post-mortem biochemical changes occur.

For metabolomic analysis, samples of meat (muscle tissue after rigor mortis) and subcutaneous adipose tissue were collected 24 h post-slaughter from the longissimus thoracis between the 12th and 13th ribs. These samples were collected during deboning, handled quickly and hygienically, snap-frozen in liquid nitrogen, and stored at −80 °C. In this context, meat refers to the same tissue after post-mortem changes, including metabolite abundance shifts that occur during the transformation from muscle to meat. From the initial 63 bulls, a subset of 15 animals, all from the same sire (*n* = 5 from each dam treatment), were randomly selected for both transcriptomic and metabolomic analyses.

### RNA extraction, processing, and sequencing

According to the manufacturer’s protocol, RNA was extracted from 100 mg of muscle tissue using the TRIzol reagent (Life Technologies, Carlsbad, CA, USA). The total RNA was quantified with the DS-11 spectrophotometer (Denovix, Wilmington, DE, USA) and its integrity was evaluated using the Bioanalyzer 2100 (Agilent, Santa Clara, CA, USA). All samples achieved an RNA integrity number (RIN) greater than 7.0, indicating good quality.

For library preparation, 0.1–1 µg of RNA was utilized following the TruSeq Stranded mRNA Reference Guide (Illumina, San Diego, CA, USA). Library quantification was conducted using quantitative PCR (35 cycles at 95 °C for 30 s) with the KAPA Library Quantification kit (KAPA Biosystems, Foster City, CA, USA), and the average library size was determined using the Bioanalyzer 2100 (Agilent, Santa Clara, CA, USA). Clustering and sequencing of the 15 samples were carried out on a single flow cell using the TruSeq PE Cluster kit v3-cBot-HS (Illumina, San Diego, CA, USA) in a paired-end approach. Sequencing was performed on the HiSeq2500 platform (Illumina, San Diego, CA, USA) with the TruSeq Stranded mRNA kit, adhering to the manufacturer’s protocol. The sequencing analysis was conducted by NGS Soluções Genômicas (Piracicaba, São Paulo, Brazil).

### Metabolomic sample processing and targeted metabolomics

Metabolites were extracted from meat and subcutaneous fat using a cold (below 0 °C with dry ice) solvent mixture of 85% HPLC-grade ethanol and 15% phosphate buffer (0.01 M, pH 7.5). Samples were weighed and homogenized three times using a bead-based homogenizer (20 s at 5500 RPM) with the extraction solvent and then centrifuged (10,000 × g for 5 min). The supernatant was collected and immediately stored in an ultra-cold freezer (−80 °C) until metabolite quantification with the AbsoluteIDQ p180 Kit. More details of the extraction protocol are available elsewhere^22^.

Apex Science (Campinas, São Paulo, Brazil) conducted the metabolomic analysis using the Biocrates Life Sciences AG AbsoluteIDQ p180 Kit, measuring 188 metabolites across several classes: amino acids, biogenic amines, acylcarnitines, lysophosphatidylcholines, phosphatidylcholines, sphingolipids, and hexose. Amino acids and biogenic amines were quantified by liquid chromatography tandem-mass spectrometry (HPLC-MS/MS). At the same time, the remaining metabolites were analyzed by flow injection analysis-tandem mass spectrometry (FIA-MS/MS). The MetIDQ software was used for data analysis, and metabolite concentrations determined using internal standards. Biocrates provides experimentally determined metabolite-specific limits of detection (LOD). Detailed information is available in^23^.

### Transcriptomics data filtering, alignment, data transformation, and normalization

Quality control analysis of raw RNA-Seq data was initially assessed using FASTQC (v. 0.11.9) (http://www.bioinformatics.babraham.ac.uk/projects/fastqc/). Adapter sequences and low-complexity reads were then removed using SeqyClean (v. 1.9.10)^24^. Cleaned reads were aligned to the *Bos taurus* ARS-UCD1.3.113 (available at: https://ftp.ensembl.org/pub/release-113/fasta/bos_taurus/) using the STAR aligner (v. 020201)^25^. Following initial processing, genes were filtered to remove those with zero counts (indicating no expression), those with low expression (averaging less than one count per million per sample), and those with fewer than ten counts in at least three samples^26,27^. The resulting count per million data was then log-transformed^28^.

### Metabolomics data filtering, scaling, and normalization

Metabolites with more than 70% of samples having values below or above the limit of detection (LOD) or with identical values across all samples were removed. This resulted in 176 metabolites for meat and 168 for subcutaneous fat. For the remaining metabolites, values below the LOD were replaced with the minimum detected value, and values above the LOD were replaced with the maximum detected value for that specific metabolite. The final dataset was then auto scaled to meet the normalization parameters. The meat and subcutaneous fat metabolomics data, as well as the metabolite annotations, are available as Additional Files 1, 2, and 3, respectively.

### WGCNA analysis

We used the Weighted Gene Co-expression Network Analysis (WGCNA) R-package v. 1.72–5.72^29^ to analyze gene co-expression and metabolite co-abundance patterns related to prenatal nutrition. The analysis also aimed to identify the hub genes and metabolites within these networks that may be central to tissue function.

For WGCNA analysis, the nutritional treatment groups (NP, PP, and FP) were converted into binary variables using dummy coding to comply with the WGCNA workflow^30^. Spearman’s correlations between gene pairs and metabolite pairs were computed separately to create the adjacency matrices. Soft thresholds were then selected to construct scale-free co-expression networks: power = 20 (R² = 0.802) for transcriptomics, power = 18 (R² = 0.622) for meat metabolomics, and power = 16 (R² = 0.910) for fat metabolomics. As the number of metabolites is often limited, the scale-free topology fit index may not reach values exceeding 0.8 for a suitable power level^29^. When the dataset does not meet the assumptions of a scale-free network, mean connectivity must be assessed^31^. Because the meat metabolomics data did not reach an R² ≥ 0.8, a soft threshold of 18 was selected (highest value of R^2^), following the protocol outlined for proteome and metabolome datasets^32^, resulting in a mean connectivity of 8.95.

Thereafter, the adjacency matrix was converted into a topological overlap matrix, followed by cluster analysis to identify modules, with a minimum of 50 genes for transcriptomics and three metabolites for metabolomics per module. Modules with a correlation (r) ≥ 0.75 were merged within each dataset. Hierarchical clustering grouped genes or metabolites with similar expression/abundance patterns into color-coded modules. Module eigengenes were calculated to summarize each module, and these eigengenes were correlated with the treatment groups (NP, PP, and FP). Gene modules were considered significant at a *P*-value < 0.05, while metabolite modules were considered significant at a *P*-value < 0.1. The less stringent threshold for metabolites was due to the smaller number of components analyzed than genes. Nevertheless, all thresholds followed the guidelines WGCNA recommended^29,32^. Heatmaps were created to visualize significant correlations between treatment groups and gene/metabolite modules. Hub genes and metabolites within significant modules were identified using the “chooseTopHubInEachModule” function in the WGCNA package.

### Functional enrichment analysis

Functional enrichment analyses were performed for genes and metabolites within each identified significant module. Gene enrichment analysis was conducted using the “enrichKEGG” function within the clusterProfiler R-package (v. 4.12.6)^33^ to identify significantly over-represented pathways using the Kyoto Encyclopedia of Genes and Genomes (KEGG) pathways database. Metabolite enrichment analysis was performed using MetaboAnalyst (v. 6.0)^34^ with the “Over Representation Analysis” function. Two separate analyses were conducted to evaluate the main metabolites sub-classes involved in each significant module: one using the RaMP-DB (Relational Database of Metabolomics Pathways; integrating KEGG via HMDB, Reactome, WikiPathways) to identify over-represented metabolic pathways, and another based on chemical structures to identify over-represented sub-chemical classes. The significance for gene and metabolite enrichments was determined by an adjusted *P*-value threshold of < 0.05.

### Data integration

For data integration, two approaches were conducted: (1) Spearman’s correlation analyses between muscle transcriptome and metabolome data (meat and subcutaneous fat) for each prenatal nutritional treatment and (2) a holistic strategy that combined all significantly associated genes and metabolites in each treatment, irrespective of the sample tissue, to assess the impact of prenatal nutrition on metabolic pathways comprehensively. For the latter, we used the “Joint Pathway Analysis” feature in MetaboAnalyst (v. 6.0), incorporating genes and metabolites identified from significantly associated modules as inputs.

If two omics elements share a common driver or one perturbs the other, they will exhibit correlation or association^35^. So, Spearman’s correlation analysis allowed us to identify muscle genes and metabolites (meat and subcutaneous fat) that are significantly correlated and consequently associated with the prenatal nutritional treatment (NP, PP, or FP). Significant correlations (adjusted *P*-value < 0.05) were visualized using heatmaps and network graphs to elucidate the relationships between transcriptome and metabolome.

The “Joint Pathway Analysis” allowed us to identify pathways underlying metabolites and genes associated with each prenatal nutritional treatment (NP, PP, and FP). Each treatment was individually analyzed to identify the metabolic pathways affected differentially according to the prenatal nutritional strategy. This analysis method relies on tight integration, combining genes and metabolites into a single query for enrichment analysis (KEGG pathway). Metabolic pathways with adjusted *P*-value < 0.05 were considered affected by the prenatal diet.

**Fig. 1:**
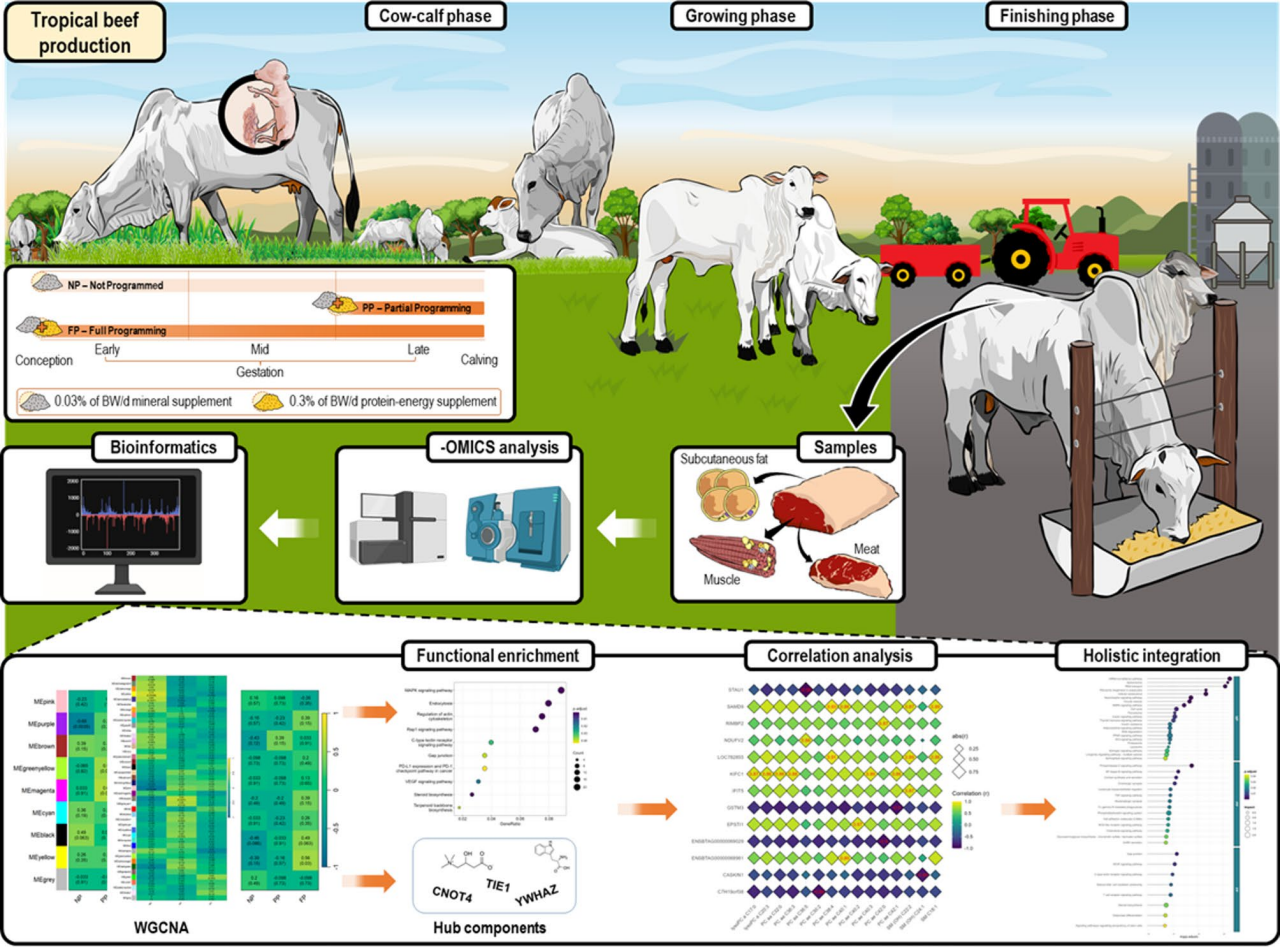
Experimental design and main bioinformatics analyses performed.

The experimental design and main analytical steps are illustrated in Fig. 1.

## Results

### WGCNA analysis

**Fig. 2:**
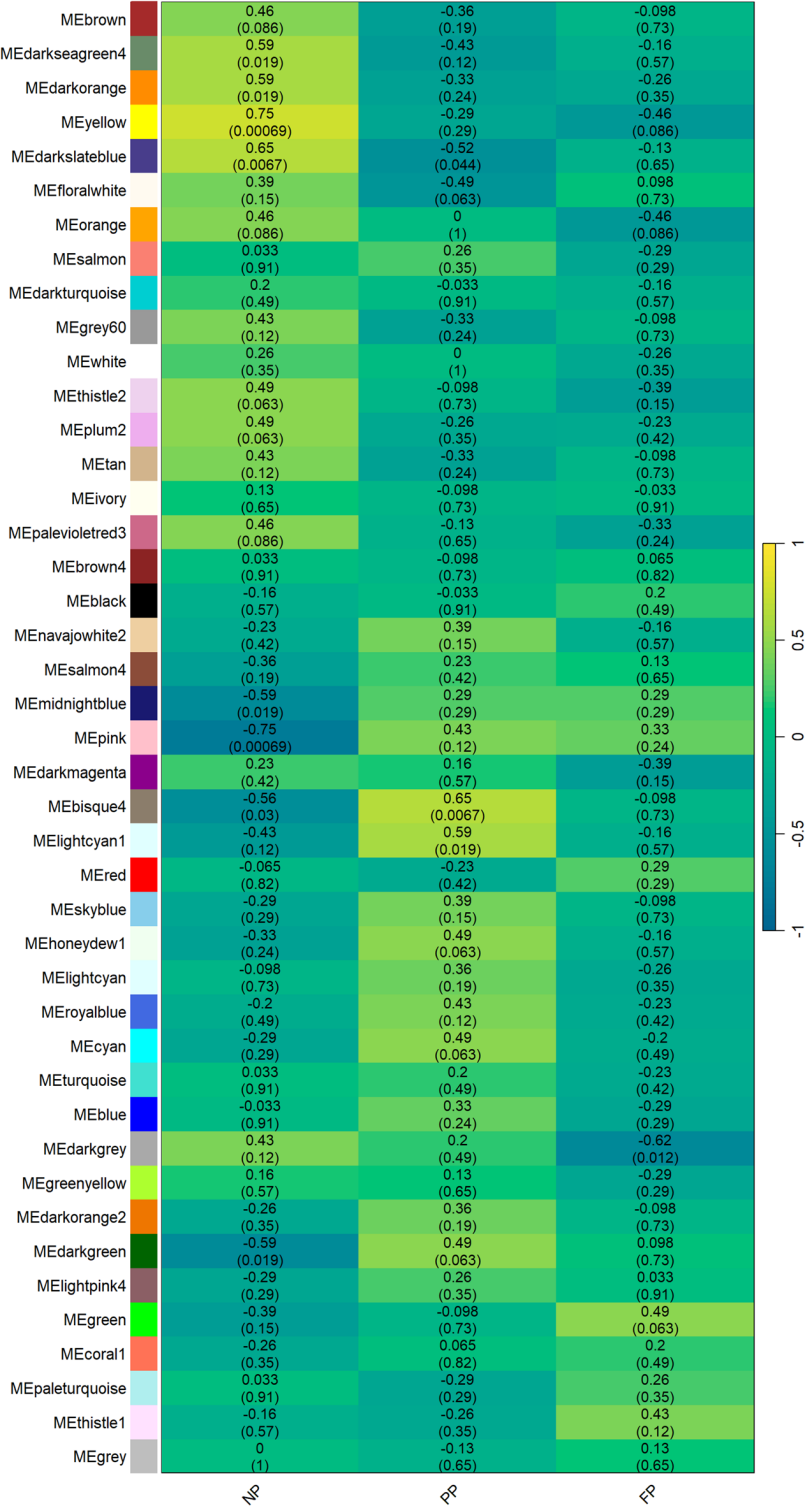
Gene module–treatment correlation heatmap. Each row corresponds to a module, and each column corresponds to a prenatal nutritional treatment group (NP, PP, and FP). Each cell contains the corresponding correlation and *P*-value. The table is color-coded by correlation, according to the color legend.

In the skeletal muscle, the gene co-expression analysis resulted in eight significantly associated modules (*p* < 0.05) for the NP group (darkseagreen4, darkorange, yellow, darkslateblue, midnightblue, pink, bisque4, and darkgreen), three significant modules for the PP (darkslateblue, bisque4, and lightcyan1), and one significant module for the FP group (darkgrey). The significant correlations (r) ranged from |0.56| to |0.75| in the NP group modules, |0.52| to |0.65| in the PP group modules, and reached − 0.62 for the darkgrey module in the FP group. As shown in Fig. 2, we identified just two shared modules between NP and PP groups (darkslateblue, and bisque4). The shared modules exhibited opposite correlation signals between the groups, indicating a different co-expression pattern in each treatment.

**Fig. 3:**
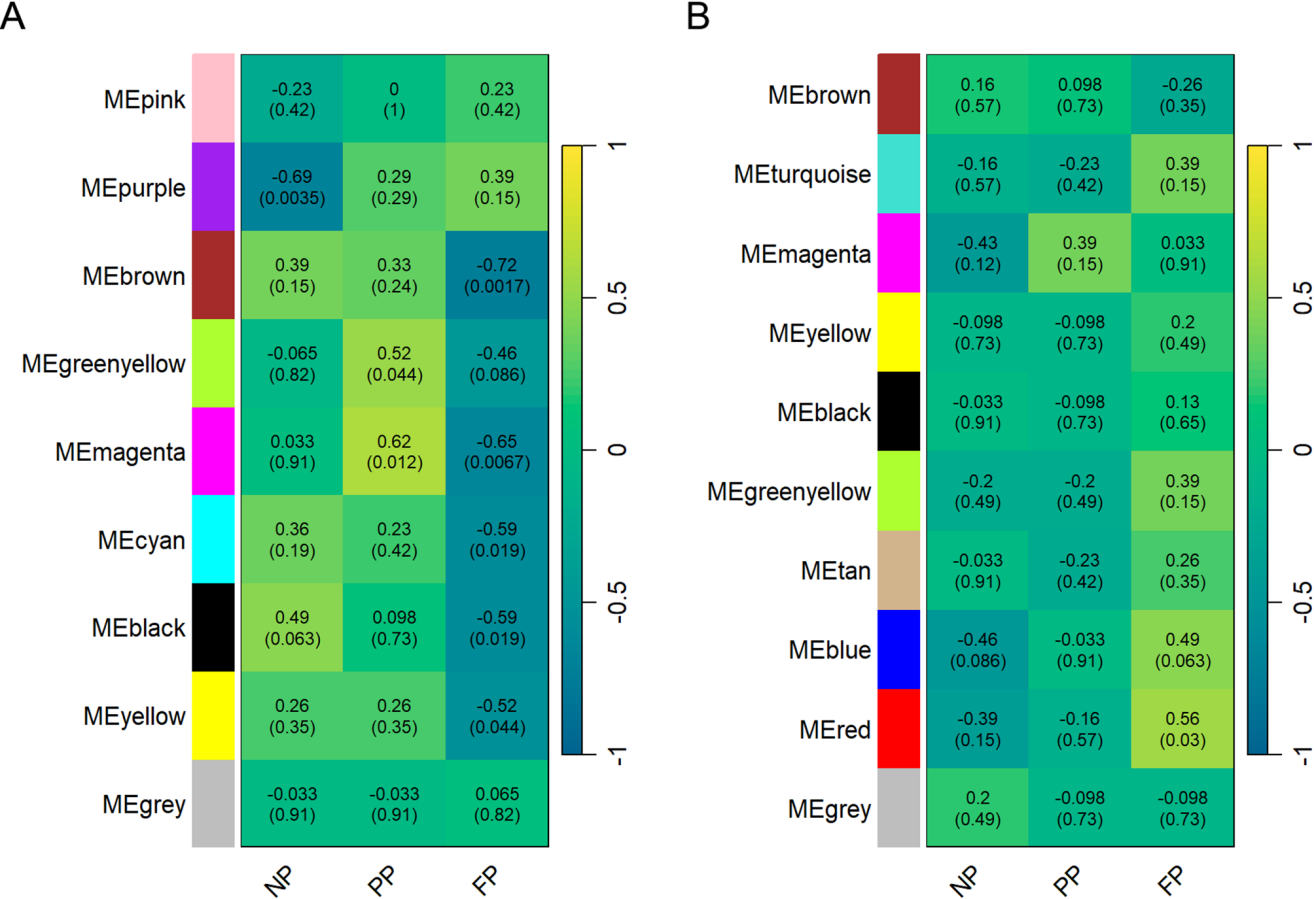
Heatmap of Metabolite Module–Treatment Correlation. Each row represents a metabolite module and each column corresponds to a prenatal nutritional treatment group (NP, PP, and FP). The values within each cell indicate the correlation coefficient and its associated *P*-value. The heatmaps are color-coded by correlation strength, according to the legend. Panel **(A)** displays the correlations for meat metabolites, while panel **(B)** represents the correlations for subcutaneous fat metabolites.

Regarding the co-abundant metabolites of meat (Fig. 3A), we found two significant modules (*p* < 0.1) for the NP group (purple and black), two for the PP (greenyellow and magenta) and six for the FP group (brown, greenyellow, magenta, cyan, black, and yellow). The correlation values ranged from |0.49| to |0.69| in the NP group, from |0.52| to |0.62| in the PP group, and from |0.46| to |0.72| in the FP group. In contrast, the co-abundance analysis of subcutaneous fat (Fig. 3B) revealed only one significant module in the NP group (blue [*r* = −0.46]), and two in the FP group (blue [*r* = 0.49] and red [*r* = 0.56]). No significant modules were identified in the PP group.

### NP group: hub genes, hub metabolites, and functional enrichment analysis

**Fig. 4:**
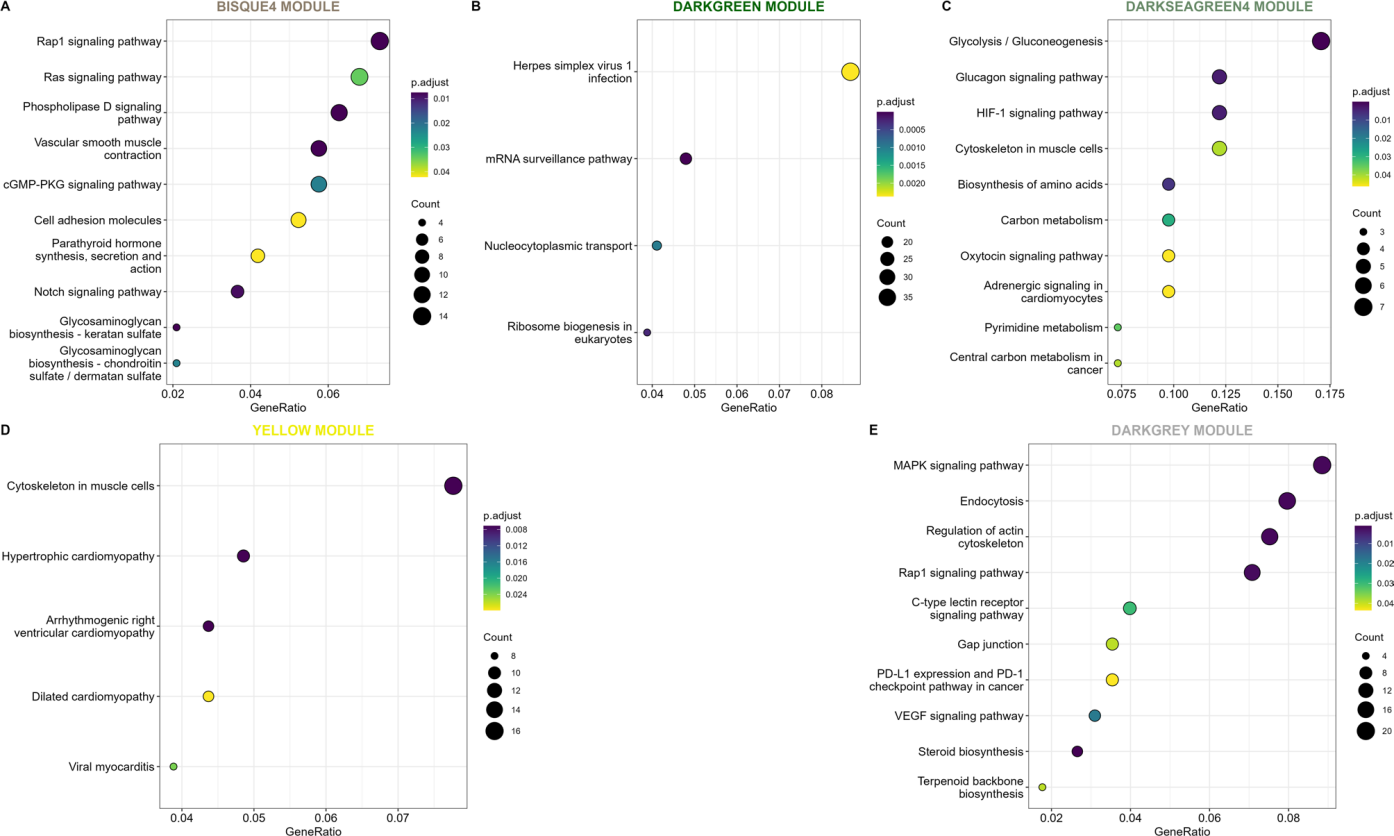
Top 10 metabolic pathways related to the genes within the significant modules across the groups (NP, PP and FP). Each letter panel corresponds to a significantly enriched module. **(A)** Bisque4 module (shared between NP and PP groups); **(B)** Darkgreen module (NP group); **(C)** Darkseagreen4 module (NP group); **(D)** Yellow module (NP group); and **(E)** Darkgrey module (FP group).

In skeletal muscle, eight hub genes were identified (Table 2): *CASQ1* (darkseagreen4), *ATP2B2* (darkorange), *ENSBTAG00000068146* (yellow), *SMYD2* (darkslateblue), *LPCAT4* (midnightblue), *CNOT4* (pink), *TIE1* (bisque4), and *RBM14* (darkgreen). Among these, the bisque4 (Fig. 4A), darkgreen (Fig. 4B), darkseagreen4 (Fig. 4C), and yellow (Fig. 4D) modules showed significant enrichment in pathways related to cellular regulation, energy metabolism, muscle growth and differentiation, hormonal responses, and cardiovascular function. These processes are essential for metabolic control, cell repair, and stress adaptation, particularly in muscle and cardiac tissue (Fig. 4).

**Table 2:** Hub genes and their gene symbols and ensembl IDs associated with the significant modules of the prenatal nutritional groups (NP, PP, and FP) identified in the WGCNA analysis.

Module	Gene Symbol	Ensembl ID
Bisque4	*TIE1*	*ENSBTAG00000003777*
Darkgreen	*RBM14*	*ENSBTAG00000001225*
Darkgrey	*YWHAZ*	*ENSBTAG00000000236*
Darkorange	*ATP2B2*	*ENSBTAG00000001305*
Darkseagreen4	*CASQ1*	*ENSBTAG00000020223*
Darkslateblue	*SMYD2*	*ENSBTAG00000013166*
Lightcyan1	*ARHGAP31*	*ENSBTAG00000000210*
Midnightblue	*LPCAT4*	*ENSBTAG00000020040*
Pink	*CNOT4*	*ENSBTAG00000017919*
Yellow		*ENSBTAG00000068146*

**Fig. 5:**
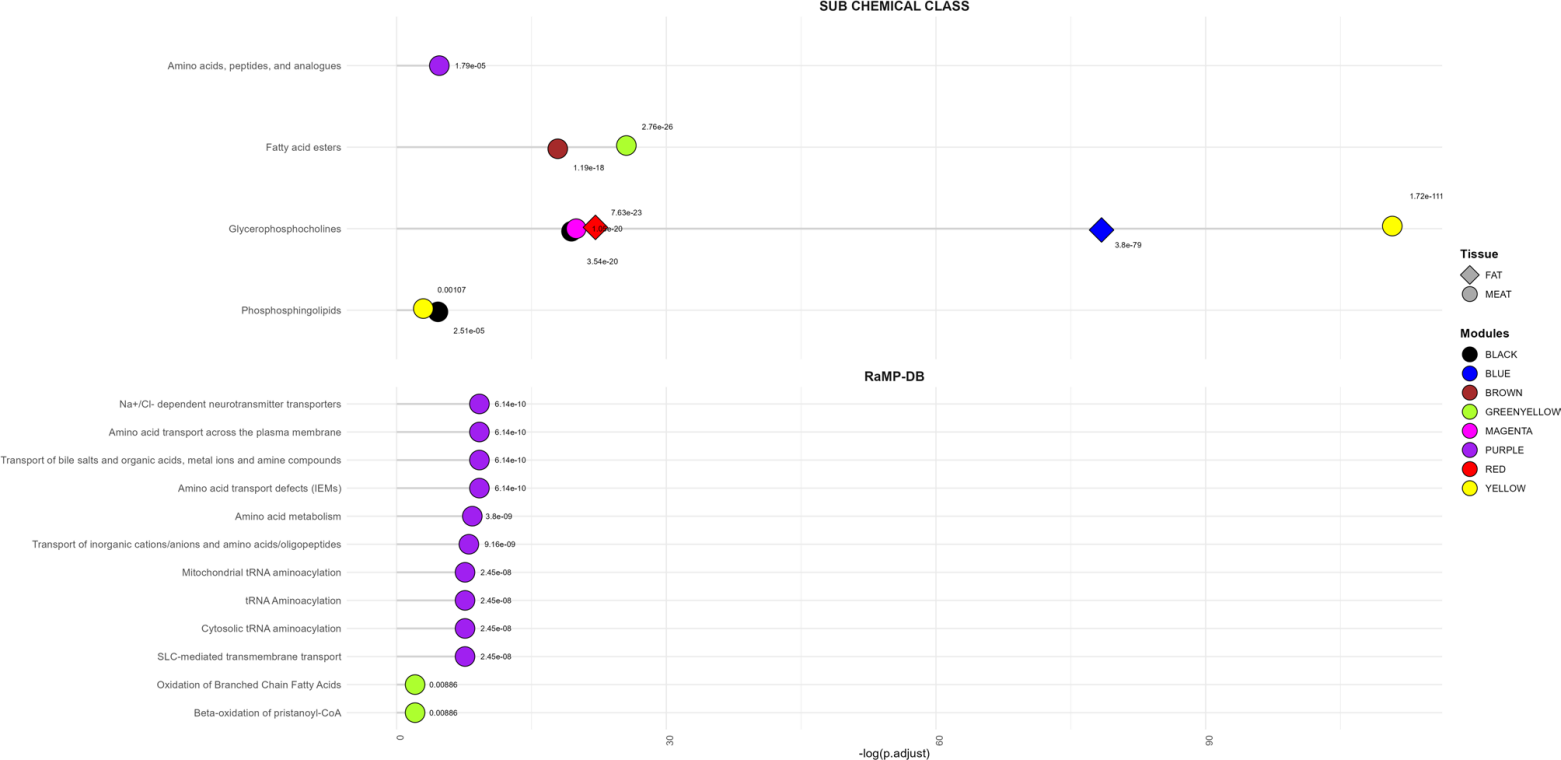
Sub-chemical class enrichment and RaMP-DB functional enrichment of each significant metabolite module. Each module is represented by a unique color, with circles indicating meat tissue and diamonds representing subcutaneous fat. The adjusted *P*-value is labeled for each significantly enriched process and subclass.

In meat (Table 3), two hub metabolites were identified: tryptophan (purple module) and sphingomyelin C24:0 (black module). The purple module was enriched in ten pathways, including Na+/Cl − dependent neurotransmitter transporters, amino acid metabolism, and multiple tRNA aminoacylation processes, highlighting its role in amino acid and neurotransmitter metabolism. The black module was enriched in glycerophosphocholines (GPCs) and phosphosphingolipids, reinforcing lipid metabolism as a major feature (Fig. 5).

**Table 3:** Hub metabolites, including their symbols and names, from significant modules identified in the WGCNA analysis of the different prenatal nutritional treatment groups (NP, PP, and FP), along with the corresponding tissues (meat and subcutaneous fat).

Tissue	Module	Metabolite symbol	Metabolite name
Meat	Black	SM C24:0	Sphingomyelin C24:0
	Brown	C5:1-DC	Glutaconylcarnitine
	Cyan	H1	Hexose
	Greenyellow	C0	Carnitine
	Magenta	PC ae C38:5	Phosphatidylcholine acyl-alkyl C38:5
	Purple	Trp	Tryptophan
	Yellow	PC ae C42:4	Phosphatidylcholine acyl-alkyl C42:4
Subcutaneous fat	Blue	PC aa C32:0	Phosphatidylcholine diacyl C32:0
	Red	PC aa C40:4	Phosphatidylcholine diacyl C40:4

In subcutaneous fat (Table 3), the blue module, present in NP and FP, contained phosphatidylcholine diacyl C32:0 as its hub metabolite, although no significant pathway enrichment was detected. Still, the module was strongly associated with the GPC sub-chemical class.

### PP group: hub genes, hub metabolites, and functional enrichment analysis

In skeletal muscle, three hub genes were identified (Table 2): *SMYD2* (darkslateblue), *TIE1* (bisque4), and *ARHGAP31* (lightcyan1). Among these, only the bisque4 module showed functional enrichment, associated with cell signaling, muscle differentiation, tissue repair, adhesion, and vascular function, all of which influence muscle development and adaptive responses (Fig. 4).

In meat, two hub metabolites were identified (Table 3): carnitine (greenyellow) and phosphatidylcholine acyl-alkyl C38:5 (magenta). The greenyellow module was enriched for fatty acid ester metabolism, particularly oxidation of branched chain fatty acids and beta-oxidation of pristanoyl-CoA, while the magenta module was enriched in GPCs (Fig. 5). No significant functional enrichment was detected for fat modules specific to PP.

### FP group: hub genes, hub metabolites, and functional enrichment analysis

In skeletal muscle (Table 2), the darkgrey module was identified with *YWHAZ* as its hub gene. This module was enriched for pathways related to cytoskeletal regulation, endocytosis, vascular function, immune modulation, and biosynthesis of steroids and terpenoids. These processes highlight its role in tissue remodeling, cellular communication, and immune–muscle interactions under prenatal programming (Fig. 4).

In meat (Table 3), six hub metabolites were identified: glutaconylcarnitine (brown), carnitine (greenyellow), phosphatidylcholine acyl-alkyl C38:5 (magenta), hexose (cyan), sphingomyelin C24:0 (black), and phosphatidylcholine acyl-alkyl C42:4 (yellow). Functionally, the brown and greenyellow modules were enriched for fatty acid esters, the magenta module for GPCs, and the yellow and black modules for both GPCs and phosphosphingolipids (Fig. 5). In subcutaneous fat (Table 3), the red module, exclusive to FP, had phosphatidylcholine diacyl C40:4 as its hub metabolite, and blue module (shared between with the NP group) phosphatidylcholine diacyl C32:0. Although not enriched in specific pathways, both red and blue modules were significantly associated with the GPC sub-chemical class (Fig. 5).

### Transcriptomic-metabolomic correlation analysis

**Fig. 6:**
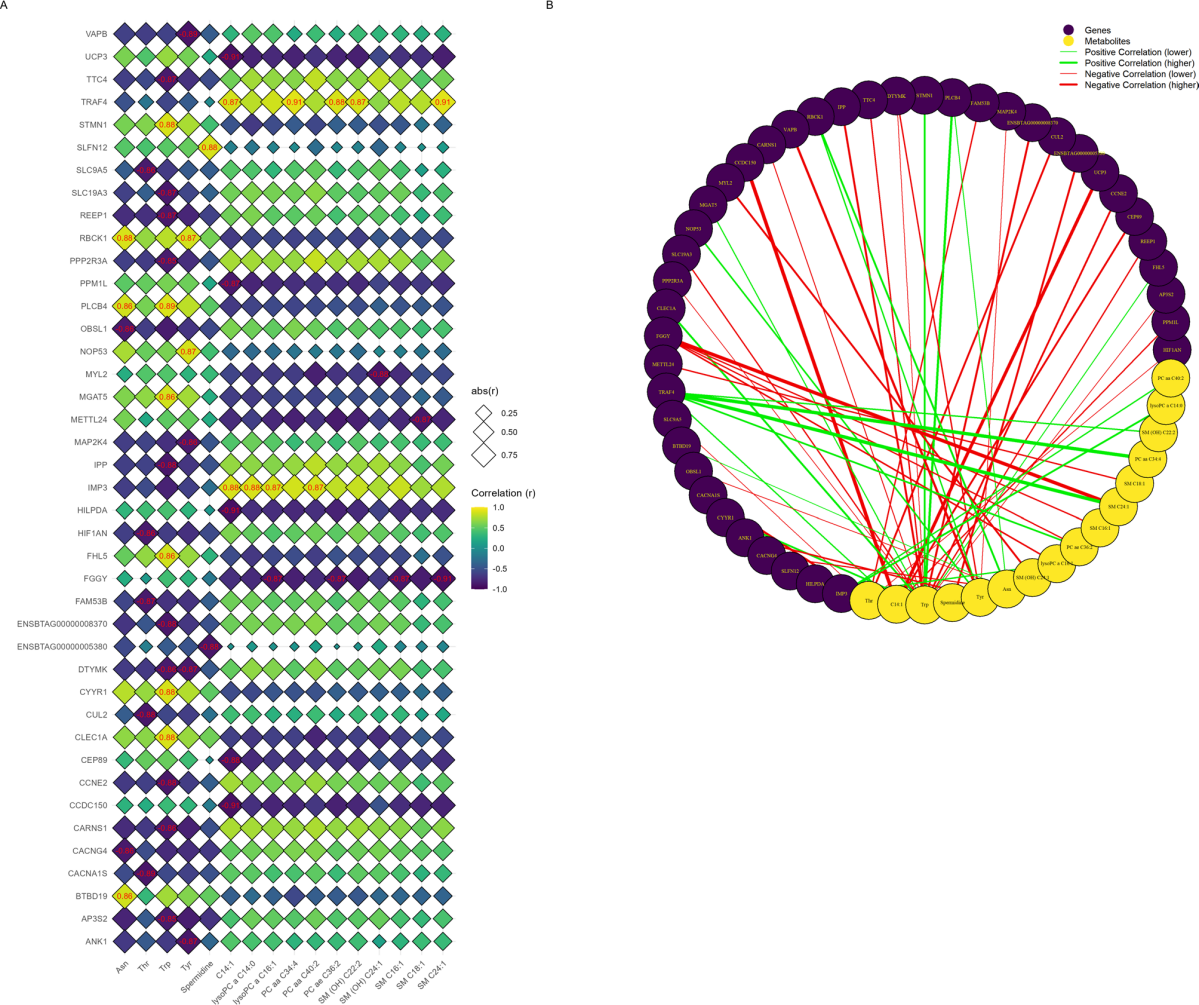
Muscle transcriptomics-meat metabolomics Spearman’s correlation analysis of the NP group. **(A)** Diamond heatmap displaying significant values, highlighted in red. The diamonds are bigger as the correlation increases in absolute value. Genes are represented on the vertical axis, while metabolites are displayed on the horizontal axis. **(B)** Correlation network graph illustrating specific connections, with red lines representing negative correlations, green lines indicating positive correlations, and thicker lines reflecting stronger correlations. Genes are represented in purple, and metabolites are shown in yellow.

Spearman’s correlation analysis between muscle genes and meat metabolites in the NP group identified 54 significant correlations (adj. *P*-value < 0.05; Fig. 6), ranging from |0.85| to |0.91|. The *TRAF4* gene exhibited the highest number of significant correlations with metabolites, with five associations identified. Conversely, tryptophan was the metabolite most strongly associated with genes, showing 16 significant correlations. Six correlations reached |0.91|: one between *TRAF4* and SM C24:1 (sphingomyelin C24:1) (*r* = 0.91), one between *TRAF4* and PC aa C34:4 (phosphatidylcholine diacyl C34:4) (*r* = 0.91), one between *FGGY* gene and SM C24:1 (*r* = −0.91), and three involving C14:1 (tetradecenoylcarnitine) with *UCP3* (*r* = −0.91), *HILPDA* (*r* = −0.91), and *CCDC150* (*r* = −0.91) genes.

**Fig. 7:**
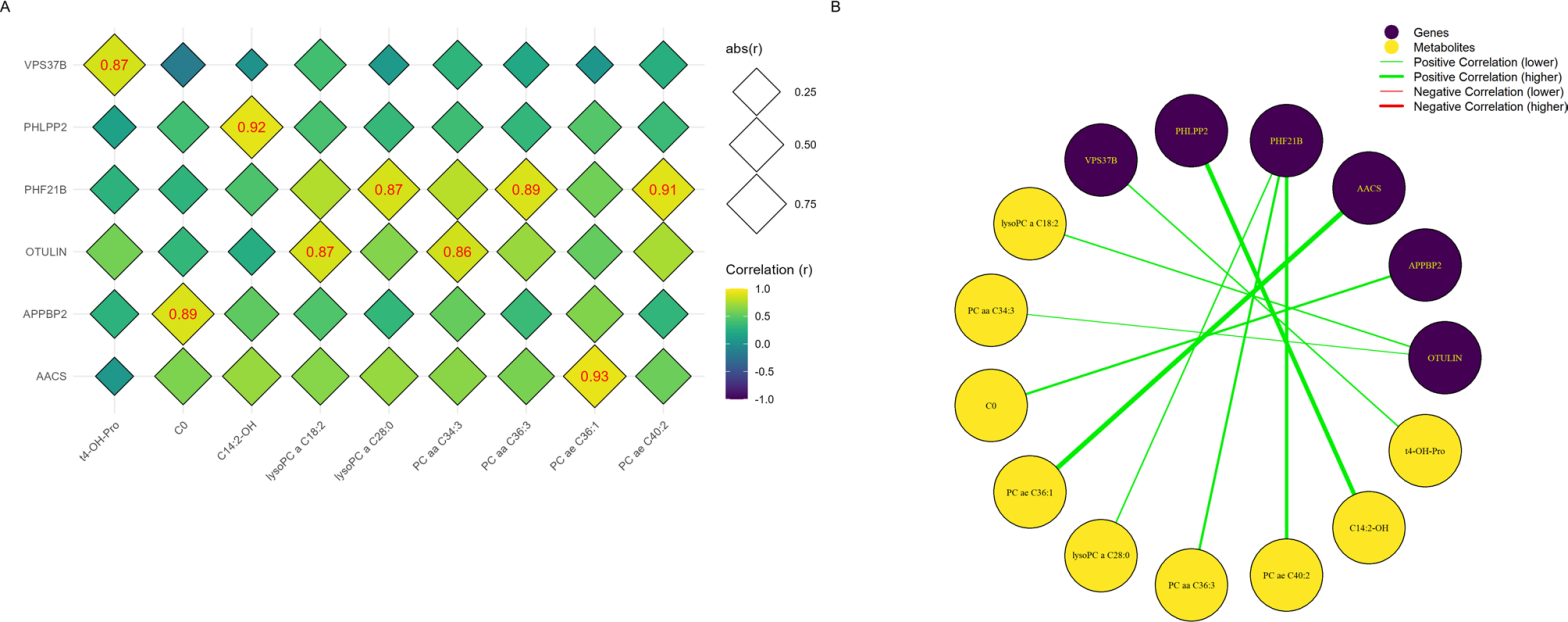
Muscle transcriptomics-meat metabolomics Spearman’s correlation analysis of the FP group. **(A)** Diamond heatmap displaying significant values, highlighted in red. The diamonds are bigger as the correlation increases in absolute value. Genes are represented on the vertical axis, while metabolites are displayed on the horizontal axis. **(B)** Correlation network graph illustrating specific connections, with red lines representing negative correlations, green lines indicating positive correlations, and thicker lines reflecting stronger correlations. Genes are represented in purple, and metabolites are shown in yellow.

Muscle-meat correlation analysis in the FP group identified nine significant correlations (Fig. 7), ranging from 0.86 to 0.93. The *PHF21B* gene showed the most significant correlations with metabolites among all genes, totaling three associations. In contrast, no single metabolite was significantly correlated with more than one gene. The strongest correlation in the FP group was observed between *AACS* gene and PC ae C36:1 (phosphatidylcholine acyl-alkyl C36:1) (*r* = 0.93). No significant correlations were found for PP group.

**Fig. 8:**
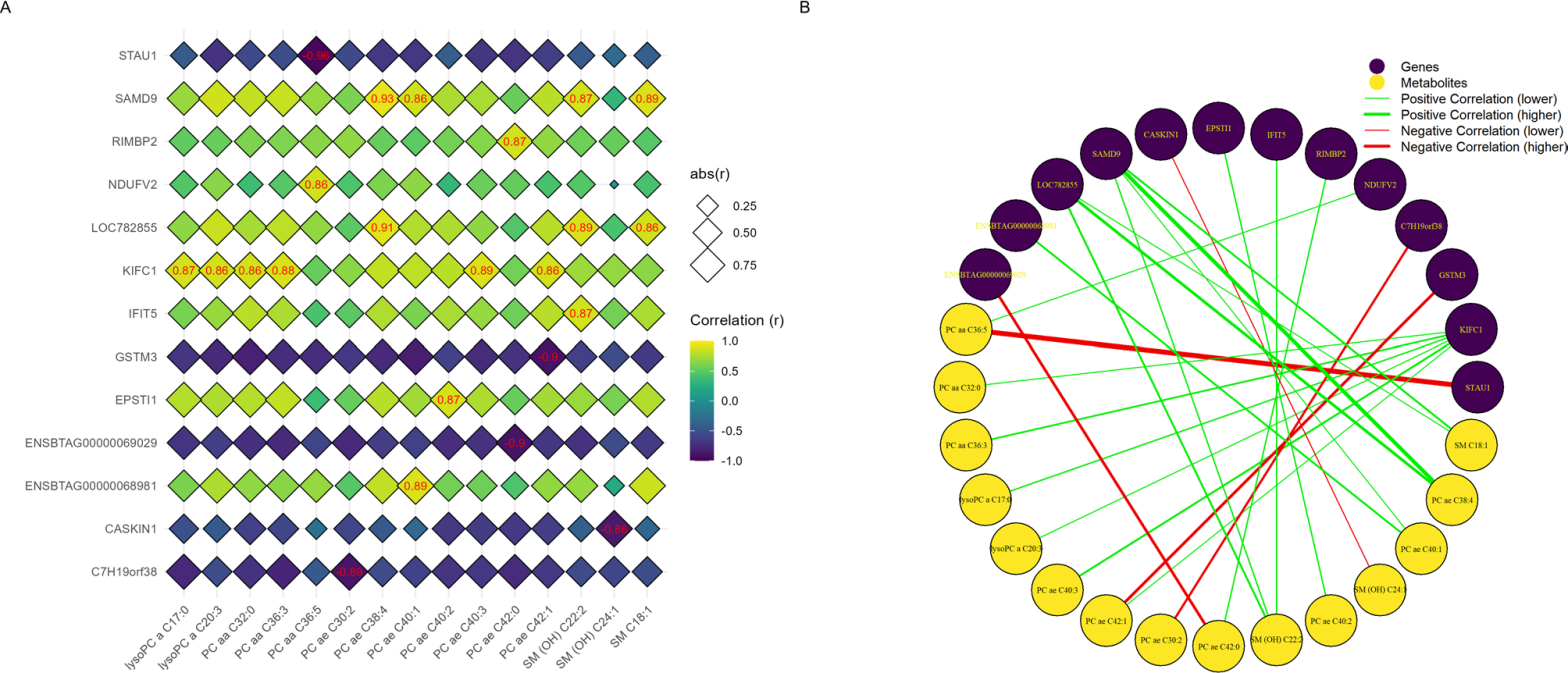
Muscle transcriptomics-fat metabolomics Spearman’s correlation analysis of the NP group. **(A)** Diamond heatmap displaying significant values, highlighted in red. The diamonds are bigger as the correlation increases in absolute value. Genes are represented on the vertical axis, while metabolites are displayed on the horizontal axis. **(B)** Correlation network graph illustrating specific connections, with red lines representing negative correlations, green lines indicating positive correlations, and thicker lines reflecting stronger correlations. Genes are represented in purple, and metabolites are shown in yellow.

Related to the correlations between significant muscle genes and fat metabolites, only significant correlations for NP group were identified (23 significant correlations; Fig. 8). The correlation coefficients ranged from |0.86| to |0.96|. Among the analyzed genes, *KIFC1* gene presented the greatest significant correlations with fat metabolites, with six associations identified. Among the metabolites, SM (OH) C22:2 (hydroxysphingomyelin C22:2) was most frequently correlated with genes, with three significant associations identified. The strongest correlation (*r* = −0.96) was identified between *STAU1* gene and PC aa C36:5 (phosphatidylcholine diacyl C36:5). No significant correlations were found for FP group.

### Transcriptomic-metabolomic integration analysis

**Fig. 9:**
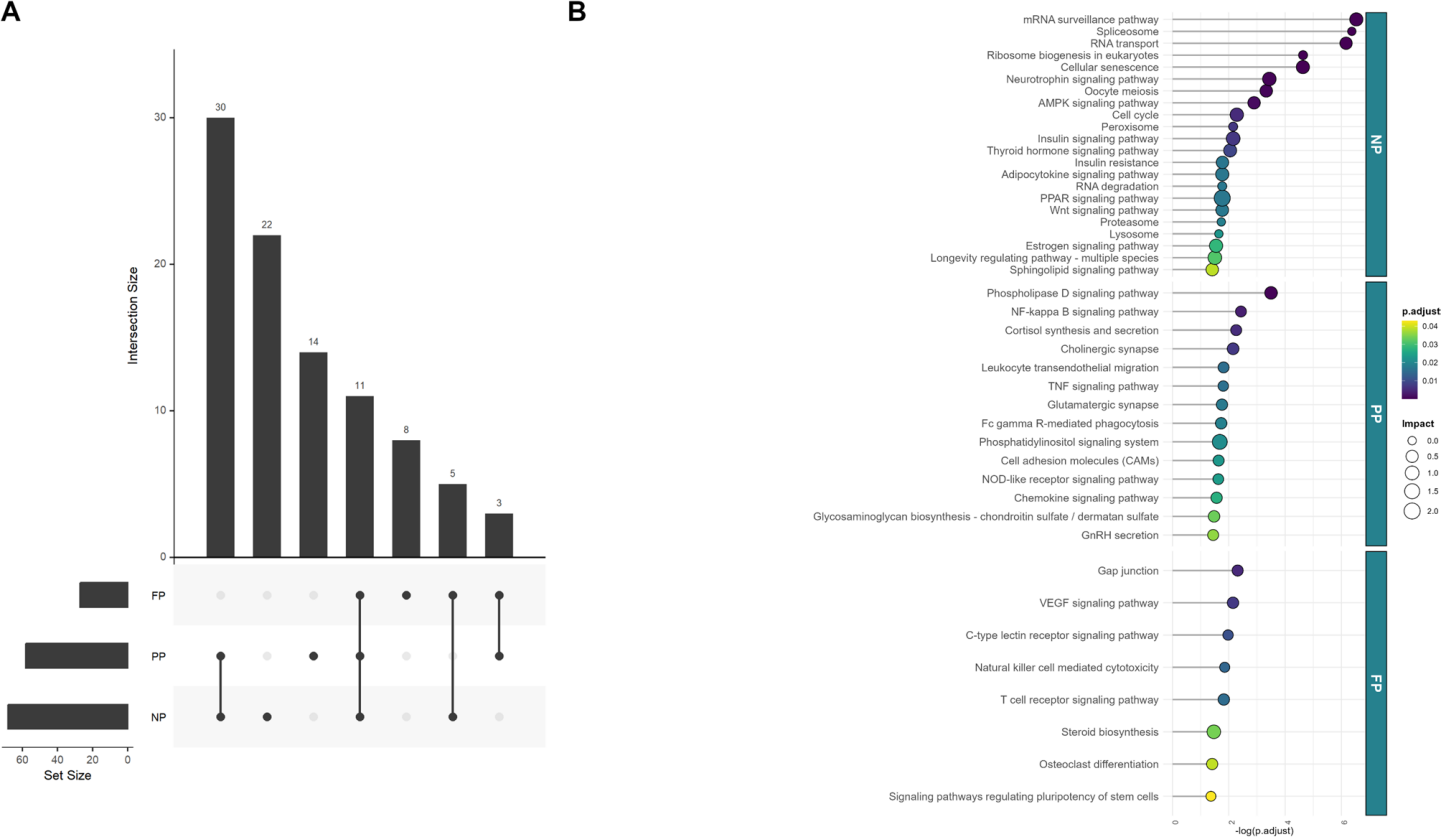
Integration analysis of significant metabolic pathways across prenatal nutritional treatments. **(A)** UpSet plot showing all significant pathways including overlapping ones identified for NP, PP, and FP groups (adjusted *P*-value < 0.05). **(B)** Lollipop chart highlighting the pathways unique to each treatment group, showing the exclusive metabolic pathways involved in NP, PP, and FP based on the transcriptomic and metabolomic integration analysis.

Based on the holistic analysis, we refined the initial set of significant pathways (adjusted *P* value < 0.05) (NP = 68, PP = 58, FP = 27; Additional File 4) and summarized all significant results, including overlapping pathways, in Fig. 9A. We then focused on pathways unique to each nutritional treatment group (NP, PP, and FP). Thus, we identified 22 distinct biological pathways for the NP group, 14 pathways associated with the PP group, and eight related to the FP group. These results are shown in Fig. 9B.

Analysis of the NP group revealed pathways intrinsically linked to meat quality and muscle characteristics. These included pathways regulating lipid metabolism (PPAR, AMPK, and sphingolipid signaling), which directly impact fat deposition, marbling, and energy balance; cellular processes like lysosome and proteasome activity, governing protein turnover and tissue remodeling; and RNA transport and ribosome biogenesis, essential for proper protein synthesis and growth. In the PP group, the analysis highlighted pathways involved in immune and inflammatory responses (e.g., TNF and NF-kappa B signaling), stress responses (e.g., cortisol synthesis), and cellular signaling (e.g., the phosphatidylinositol signaling system and GnRH secretion), suggesting a potential influence on tissue development and stress adaptation. The FP group exhibited pathways associated with nutrient delivery (VEGF signaling), hormonal regulation (steroid biosynthesis), intercellular communication (gap junctions), and tissue differentiation (stem cell signaling), all of which play a role in shaping muscle growth, fat deposition, and ultimately, meat quality.

## Discussion

The Discussion section has been divided into subsections, each addressing a specific prenatal nutrition group (NP, PP, and FP), followed by a subsection dedicated to the interconnections among them (Sect. “How do the prenatal nutrition groups (NP, PP and FP) interrelate?”). Throughout these subsections, we highlight the key differences observed between the groups, emphasizing the holistic nature of the findings. Finally, we added a dedicated subsection on the limitations of the study to acknowledge potential constraints and to guide future research directions. Our primary objective is to discuss these integrated results, as combining multiple omics approaches yield more robust, trustworthy and accurate conclusions^35–37^.

### NP group predominantly involved in cellular processes, protein dynamics, and energy metabolism

Most of the correlations found in the NP group are classified as moderate^38^; however pink (*r* = −0.75) and yellow (*r* = 0.75) modules were strongly correlated with NP group. Although all metabolite modules exhibited moderate correlations (meat and subcutaneous fat), the meat purple module stood out by presenting the strongest correlation with the NP group (*r* = −0.69).

The hub gene *CNOT4* (pink module), part of the CCR4-NOT complex, regulates gene expression, DNA repair, and protein ubiquitination^39–41^, but its role in skeletal muscle or prenatal nutrition in beef cattle is not yet established. The hub gene of the yellow module (*ENSBTAG00000068146*) has not been fully annotated. Nevertheless, this module is significantly enriched for cytoskeletal processes in muscle cells. The cytoskeleton, composed of actin, microtubules, and intermediate filaments, coordinates cellular signaling, influencing muscle growth, contraction, and regulation of signaling pathways^42^. Moreover, cytoskeletal proteins influence DNA repair, genome stability, gene expression, and mRNA translation^43–46^. In beef cattle, cytoskeletal proteins and transmembrane anchors are important for meat quality by maintaining cytoskeletal structure and integrity postmortem^47^. Notably, the “cytoskeleton in muscle cells” pathway was enriched in both the darkseagreen4 and yellow modules, exclusively associated with the NP group, suggesting that prenatal nutrition may impact muscle and/or meat properties. However, our previous study did not reveal substantial alterations in the assessed meat quality parameters^6^.

Regarding metabolite modules, the purple module in meat was strongly associated with amino acid metabolism, including several related pathways, with tryptophan as the hub component. Tryptophan also had the highest number of correlations in the integrative transcriptome–metabolome analysis of the NP group, highlighting its pivotal role. Supplementation of tryptophan has been shown to enhance antioxidant capacity and meat quality in ruminants, potentially by reducing stress^48^. Additionally, dietary tryptophan may increase serum glucose, insulin, and IGF-I levels, influencing protein metabolism via insulin-mediated nutrient assimilation^49^.

The holistic analysis of the NP group revealed several pathways associated with cellular processes, consistent with the individual single -OMIC findings. Three key metabolic pathways were particularly relevant to prenatal nutrition and meat quality: the PPAR signaling pathway, the Wnt signaling pathway, and the sphingolipid signaling pathway. Previous studies by Diniz et al.^50,51^ showed that maternal vitamin/mineral supplementation and weight gain affected PPAR signaling in placenta and fetal liver; our study extends these findings, demonstrating lasting effects on offspring energy metabolism via PPAR signaling up to market age. On the other hand, the Wnt signaling pathway plays a key role in muscle development during both fetal and adult stages. In adulthood, canonical Wnt signaling is associated with satellite cell differentiation^52^, regulates energy metabolism by stimulating glucose uptake via Akt1 and AMPK^53^ and modulate genes involved in glycemic homeostasis and mitochondrial energy expenditure^54^. In our study, insulin resistance–related pathways were activated in skeletal muscle of NP animals. Thus, Wnt signaling may serve as an alternative mechanism for insulin-independent glucose uptake, particularly in intramuscular adipose tissue^55^, which may explain the absence of differences in total lipid content and marbling scores reported in a previous study performed with the same animals^6^.

Additionally, we identified the regulation of *UCP3* in the skeletal muscle of the NP group, a gene associated with mitochondrial uncoupling and increased energy expenditure. Although *UCP3* is not directly linked to the Wnt pathway, its expression may be influenced by metabolic processes mediated by Wnt signaling, such as mitochondrial biogenesis and oxidative phosphorylation^54^. Thus, it is plausible that lower metabolic efficiency and greater energy expenditure in the skeletal muscle of NP animals, when fed high-energy diets typical of feedlot finishing, may have contributed to the reduced subcutaneous fat deposition observed in a previous study with the same animals^6^. This hypothesis is further supported by transcriptomic-metabolomic evidence linking sphingolipid metabolism to regulating subcutaneous adiposity in beef cattle^56^ considering that this pathway may induce insulin resistance^57^.

### PP group: primarily involved in nutrient delivery, immune processes, stress response, and cellular signaling

Among the three gene modules significantly associated with the PP treatment, the bisque4 module exhibited the strongest correlation (*r* = 0.65). The hub gene within this module, *TIE1* gene, encodes a transmembrane tyrosine kinase predominantly expressed in endothelial cells. The *TIE1* gene plays a crucial role in angiogenesis and vascular stability^58^. Interestingly, significant pathways identified in this module, including Rap1, Ras, and Notch signaling pathways, are closely related to angiogenesis and vascular remodeling. The Rap1 and Ras pathways are integral to endothelial cell migration and proliferation^59^, while Notch signaling pathway regulates vascular branching and stabilization^60^, processes crucial for vessel formation. Angiogenesis is essential for muscle development, as the formation of new blood vessels provides the necessary supply of oxygen and nutrients to growing muscle tissues^61^.

Co-abundance analysis of the metabolome data identified only two significant modules within the PP group, both associated with meat. The magenta module exhibited the strongest correlation (*r* = 0.62) with the PP treatment. PC ae C38:5 was identified as the hub metabolite within this module, and the sub-chemical class associated with this module aligned with the GPC class to which the hub metabolite belongs.

The GPCs are a crucial class of lipids involved in numerous physiological functions. They are fundamental components of cellular membranes, participating in signaling pathways and metabolic processes^62^. They also regulate lipid metabolism, cellular volume (osmoregulation), and overall cellular balance^63,64^. In skeletal muscle, GPCs act as key osmolytes, maintaining intracellular water content during osmotic stress, such as intense exercise, which is essential for preserving muscle cell structure^65^. Notably, our previous study has reported greater cooking loss in animals from the PP group, suggesting that their muscle tissue may have retained more water in vivo, leading to higher water loss during cooking^6^.

The holistic analysis confirmed the significance of pathways previously identified in single-omic over-representation analyses, including Glycosaminoglycan biosynthesis – chondroitin sulfate/dermatan sulfate and the Phospholipase D signaling pathway. Glycosaminoglycans are complex polysaccharides important for growth, differentiation, morphogenesis, cell migration, and responses to bacterial/viral infections^66^. Phospholipase D hydrolyzes phosphatidylcholine, generating phosphatidic acid (PA), a key signaling lipid involved in cellular processes such as signaling, membrane dynamics, and metabolism. PA can be further metabolized into bioactive lipids like diacylglycerol and lysophosphatidic acid, expanding its role in cell growth, cytoskeletal remodeling, and lipid metabolism regulation^67^. Phospholipase D activity is also linked to hormonal and stress signaling pathways^68^, which are closely associated with the GPC sub-class enriched in the PP group.

Other identified pathways are related to immune system function and stress responses, such as NF-kappa B signaling and cortisol synthesis and secretion. NF-kappa B transcription factors are pivotal in regulating immune and inflammatory responses^69^, essential for expression of pro-inflammatory cytokines, chemokines, and adhesion molecules^70^. NF-kappa B signaling has been implicated in various immune responses to diseases in bovine species^71–73^. Regarding the cortisol pathway identified, several studies have associated it with beef cattle production traits^74,75^ and fetal programming in ruminants^76–78^. Interestingly, in our previous liver integrative study^18^, we found some pathways linked with immune and stress functions in the protein-energy supplementation groups (PP and FP), confirming consistency across tissues.

The involvement of the NF-kappa B signaling pathway highlights its critical role in regulating immune and inflammatory responses, which are essential for maintaining health and resilience in bovine species. Meanwhile, the cortisol synthesis and secretion pathway underscore its influence on key production traits, including stress resilience, growth, and development, as well as its long-term effects through fetal programming.

### FP group is significantly associated with nutrient delivery, steroid biosynthesis, and immune response

Among gene modules, only the darkgrey module was significantly associated with the FP group (*r* = −0.62). In contrast, the metabolomic analyses identified eight significant modules, with the strongest correlation observed in the meat brown module (*r* = −0.72).

The darkgrey hub gene, *YWHAZ*, is a member of the 14-3-3 gene family, which encodes a highly conserved group of molecular chaperones involved in cell cycle regulation, proliferation, apoptosis, invasion, migration, and neuronal development^79–81^. The functional enrichment of this module revealed important pathways most related to immune process (MAPK signaling, endocytosis, and C-type lectin receptor signaling), cellular communication (gap junction), hormone synthesis (steroid biosynthesis), and nutrient delivery (VEGF signaling pathway). The MAPK signaling pathway, a key component of this module, has been previously implicated in amino acid supplementation^82^. This finding suggests a potential link between the MAPK pathway and the prolonged protein-energy supplement provided to FP dams in the present study, which may have multigenerational consequences for the offspring. Interestingly, Diniz et al.^83^ demonstrated that prenatal nutrition in beef cattle influenced the MAPK signaling pathway in muscle and liver tissues of offspring at day 50 of gestation, supporting the relevance of this pathway in nutritional and developmental contexts.

The meat brown module hub metabolite, glutaconylcarnitine, an acylcarnitine derivative, facilitates transport of fatty acids into mitochondria for β-oxidation, supporting cellular energy production^84^. This aligns with the over-representation of fatty acid esters (sub-class related to lipid metabolism) within the same module. Fatty acid esters are critical intermediates in lipid storage and mobilization and are highly responsive to maternal nutrition during gestation. Studies in beef cattle have demonstrated that prenatal nutrition can significantly influence the offspring’s lipid metabolism, potentially altering fat deposition in intramuscular and subcutaneous fat depots, both of which are key determinants of meat quality^10,85^. For instance, maternal dietary interventions rich in energy have been shown to modulate lipid profiles in progeny^86^, which can impact in traits important for meat tenderness and flavor. However, there is still limited information on the effects of prenatal nutrition on the fatty acid profile of meat from offspring^87^. The central role of glutaconylcarnitine in fatty acid oxidation underscores its significance in linking prenatal supplementation to metabolic programming in offspring, with implications for improving economic and nutritional value of beef.

Holistic pathway analysis revealed an over-representation of several pathways previously enriched in the darkgrey gene module, including gap junctions, VEGF signaling, C-type lectin receptor signaling, and steroid biosynthesis, further emphasizing their relevance to the FP group. Gap junctions play a critical role in intercellular communication, enabling the transfer of ions and signaling molecules between adjacent cells to ensure coordinated cellular responses and tissue homeostasis^88^. The VEGF signaling pathway is essential for angiogenesis, which promotes vascular development and nutrient delivery, directly influencing tissue growth and development^89^. The C-type lectin receptor signaling pathway is crucial for immune recognition and response, facilitating the detection of pathogens and maintaining organismal health^90^. Steroid biosynthesis supports the production of key hormones that regulate metabolism, reproduction, and stress responses, which are critical for overall physiological balance^91^. Collectively, these pathways highlight the biological processes modulated by the FP group, with potential implications for both metabolic efficiency and immune competence in the progeny.

### How do the prenatal nutrition groups (NP, PP and FP) interrelate?

While some metabolic pathways, gene co-expression, and metabolite co-abundant modules were shared across prenatal groups, the correlation signals and magnitudes varied. For example, the bisque4 module showed opposite correlations with NP (*r* = −0.56) and PP (*r* = 0.65), suggesting that shared pathways may function through distinct mechanisms in each group.

PP and FP groups shared immune- and stress-related pathways, including NF-kappa B and C-type lectin receptor signaling, as well as angiogenesis-related pathways (Rap1, Ras, Notch, and VEGF), reflecting convergent adaptive responses to enhanced maternal nutrition.

Despite being more pronounced in the NP group, our findings demonstrate a substantial impact of prenatal nutrition on lipid metabolism, as evidenced by the enrichment of a significant portion (87.5%) of metabolite modules with lipid sub-classes. Notably, the only module related to amino acid metabolism was exclusively associated with the NP group (purple module), suggesting a unique influence of this nutritional group on protein metabolism in the offspring. This finding is particularly relevant in the context of beef production, where both lipid and protein metabolism significantly influence the quality and composition of beef in cattle. While further research is needed to fully elucidate the specific effects of prenatal nutrition on each metabolic pathway, this study provides significant insights into the field of fetal programming in beef cattle. By uncovering the underlying molecular mechanisms associated with meat-relevant tissues, these findings contribute to a better understanding of how maternal nutrition shapes offspring development and meat quality.

### Limitations of the study

This study has some limitations that should be acknowledged. First, the relatively small sample size for transcriptomic and metabolomic analyses (*n* = 5 per group, totaling 15 animals) may limit statistical power and the generalizability of the findings. However, according to WGCNA guidelines, this sample size is adequate for detecting robust co-expression modules in exploratory datasets. Still, the reduced number of animals constrains our ability to identify more subtle molecular differences and limits inferences about causality. Second, we did not perform experimental validation of transcriptomic findings, such as qPCR confirmation of hub genes, and while targeted metabolomics was conducted using HPLC-MS/MS and FIA-MS/MS, providing reliable quantification of metabolites, transcriptomic results should be interpreted as indicative. Additionally, correlations identified through multi-omics integration cannot establish causal relationships. Nonetheless, the combination of muscle transcriptomics with targeted metabolomics from fat and meat provided cross-tissue consistency that strengthens confidence in the major biological patterns detected. To the best of our knowledge, this is the first integrative multi-omics study focusing on specific tissues directly linked to meat quality in the context of long-term prenatal nutritional effects in beef cattle. Overall, this multi-omics framework offers valuable exploratory insights into the effects of prenatal nutrition, but future studies with larger cohorts and targeted validation assays are required to confirm and extend these results.

## Conclusions

Our study provides exploratory insights into the impact of prenatal nutrition on key metabolic pathways and hub components in bovine skeletal muscle, meat, and subcutaneous fat. The results suggest that distinct patterns emerge across dietary interventions, with all treatments affecting energy metabolism and cellular processes. The NP group was more strongly associated with protein metabolism and showed the highest number of pathways exclusively enriched for lipid metabolism, suggesting a potential influence on this metabolic process. Protein-energy-supplemented groups (PP and FP) were more closely associated with pathways related to immune function, stress resilience, and nutrient delivery. Interestingly, some shared modules between groups exhibited inverse correlations, which may reflect antagonistic effects or epigenetic modifications driven by prenatal nutrition. Overall, these findings highlight critical metabolic pathways relevant to meat production and provide a foundation for future studies to further explore the role of prenatal nutrition in animal health and productivity.

## Supplementary Information

Below is the link to the electronic supplementary material.

Supplementary Material 1

## Data Availability

The RNA-seq data used for the current study is available in the European Nucleotide Archive (ENA) repository (EMBL-EBI), under accession PRJEB84398 (http://www.ebi.ac.uk/ena/browser/view/PRJEB84398. The metabolomic data for meat and subcutaneous fat analyzed in this study are provided in Additional files 1 and 2, respectively. All additional files are available in Supplementary Material 1.

## Abbreviations

AACS: acetoacetyl-CoA synthetase
ARHGAP31: Rho GTPase Activating Protein 31
ATP2B2: ATPase Plasma Membrane Ca2 + Transporting 2
BW: Body weight
C14: 1:tetradecenoylcarnitine
C14: 2-OH: hydroxytetradecadienylcarnitine
CASQ1: Calsequestrin 1
CCDC150: Coiled-Coil Domain Containing 150
CNOT4: CCR4-NOT Transcription Complex Subunit 4
CP: Crude protein
DMI: Dry matter intake
FGGY: FGGY Carbohydrate Kinase Domain Containing
FIA-MS/MS: Flow injection analysis-tandem mass spectrometry
FP: Full programming
GPC: glycerophosphocholines
HILPDA: Hypoxia Inducible Lipid Droplet Associated
HPLC: High-performance liquid chromatography
HPLC-MS/MS: Liquid chromatography tandem-mass spectrometry
IEMs: amino acid transport defects
KEGG: Kyoto encyclopedia of genes and genomes
KIFC1: Kinesin Family Member C1
LOD: Limits of detection
LPCAT4: Lysophosphatidylcholine Acyltransferase 4
MAPA: Ministry of Agriculture, Livestock, and Supply of Brazil
NDF: Neutral detergent fiber
NP: Not programmed
PA: phosphatidic acid
PC aa C34: 4:phosphatidylcholine diacyl C34:4
PC aa C36: 5:phosphatidylcholine diacyl C36:5
PC ae C36: 1:phosphatidylcholine acyl-alkyl C36:1
PHF21B: PHD Finger Protein 21B
PP: Partial programming
r: correlation
RBM14: RNA Binding Motif Protein 14
RIN: RNA integrity number
SM (OH) C22: 2:hydroxysphingomyelin C22:2
SM C24: 1:sphingomyelin C24:1
SMYD2: SET and MYND Domain Containing 2
TDN: Total digestible nutrients
TIE1: Tyrosine Kinase with Immunoglobulin Like and EGF Like Domains 1
TRAF4: TNF Receptor-Associated Factor 4
UCP3: Uncoupling Protein 3
WGCNA: Weighted gene co-expression network analysis
YWHAZ: Tyrosine 3-Monooxygenase/Tryptophan 5-Monooxygenase Activation Protein Zeta
